# Upregulation of miRNA hsa-miR-342-3p in experimental and idiopathic prion disease

**DOI:** 10.1186/1750-1326-4-36

**Published:** 2009-08-27

**Authors:** Judith Montag, Reiner Hitt, Lennart Opitz, Walter J Schulz-Schaeffer, Gerhard Hunsmann, Dirk Motzkus

**Affiliations:** 1 Department of Infection Biology, German Primate Center, Kellnerweg 4, 37077 Goettingen, Germany; 2 Center 3 Biochemistry and Molecular Cell Biology, DNA Microarray Facility, Humboldtallee 23, 37073 Goettingen, Germany; 3 Prion and Dementia Research Unit, Department of Neuropathology, University of Goettingen, Robert-Koch-Str. 40, 37075 Goettingen, Germany; 4 Department of Virology and Immunology, German Primate Center, Kellnerweg 4, 37077 Goettingen, Germany; 5 Department of Infection Models, German Primate Center, Kellnerweg 4, 37077 Goettingen, Germany

## Abstract

The aim of our study was to analyze the differential expression of miRNAs in the brains of BSE-infected cynomolgus macaques as a model for Creutzfeldt-Jakob disease (CJD). MicroRNAs (miRNAs) are small noncoding RNAs regulating gene expression by mRNA targeting. Among other functions they contribute to neuronal development and survival. Recently, the lack of miRNA processing has been shown to promote neurodegeneration and deregulation of several miRNAs has been reported to be associated with Scrapie in mice. Therefore, we hypothesized that miRNAs are also regulated in response to human prion disease. We have applied miRNA-microarrays to identify deregulated miRNA candidates in brains of BSE-infected macaques. Shock-frozen brain sections of six BSE-infected and five non-infected macaques were used to validate regulated miRNA candidates by two independent qRT-PCR-based methods. Our study revealed significant upregulation of hsa-miR-342-3p and hsa-miR-494 in the brains of BSE-infected macaques compared to non-infected animals. In a pilot study we could show that hsa-miR-342-3p was also upregulated in brain samples of human type 1 and type 2 sporadic CJD. With respect to the reported regulation of this miRNA in Scrapie-infected mice, we propose that upregulation of hsa-miR-342-3p may be a general phenomenon in late stage prion disease and might be used as a novel marker for animal and human TSEs.

## Findings

Transmissible spongiform encephalopathies (TSE) comprise a group of neurodegenerative diseases including bovine spongiform encephalopathy (BSE) in cattle, Scrapie in sheep, and Creutzfeldt-Jakob disease (CJD) in humans [1]. The causative agent is or is closely related to a pathogenic isoform of the cellular prion protein (PrP^c^). It is generally assumed that the alpha-helix-rich isoform changes conformation to form the beta-sheet-rich counterpart, PrP^Sc ^[2]. Aggregation of PrP^Sc ^coincides with neuronal loss that finally leads to death of the host. The molecular mechanism underlying prion-induced neurodegeneration is still poorly understood.

Several lines of evidence indicate that microRNAs (miRNAs) may play a pivotal role in neurodegeneration [3,4]. MiRNAs are derived from primary transcripts (pri-miRNA) that are sequentially processed into their mature form by the RNase III type nucleases DROSHA and DICER [5]. Incorporation of miRNAs in RNA-induced silencing complexes (RISCs) then leads to decay or to translational repression of complementary mRNA targets.

Loss of DICER activity has been linked to cerebellar degeneration, neurotoxicity, and ataxia in *Drosophila *and mice [6,7]. Furthermore, a role for miRNAs was also described for neurodegenerative diseases in humans, such as Morbus Parkinson [8], Chorea Huntington [9] and Alzheimer's disease [10]. Most recently, the regulation of endogenous miRNAs has also been linked to animal prion disease [11]. Thus we hypothesized that miRNA regulation may also play a role in human prion diseases. To investigate whether prion-induced neurodegeneration is linked to deregulation of miRNA in the brain of affected individuals, we analyzed differential miRNA expression in brains of BSE-infected non-human primates (*Macaca fascicularis*) as a model for Creutzfeldt-Jakob disease in humans.

Previous studies have shown that the clinical course of disease and the lesion profile in the central nervous system of BSE-infected macaques are comparable to acquired human CJD [12]. The tissues used for our study were derived from six age- and sex-matched *M. fascicularis *intracerebrally infected with brain homogenate from BSE-infected cattle. Upon disease progression, BSE-affected macaques displayed ataxia and tremors as first signs of neuronal defects. Euthanasia was indicated by three or more of the following clinical observations: loss of hand-eye coordination, dehydration, myoclonus, apathy. PrP^Sc ^aggregates in the brains of BSE-infected macaques were confirmed using biochemical and immunohistochemical methods according to established protocols [13]. Detailed results of the on-going risk assessment study will be reported elsewhere.

For the identification of deregulated miRNAs we applied miRNA microarrays which have been widely used to analyze miRNA expression patterns. For our approach we have used shock-frozen biopsy punches of the *basis pontis *region of one BSE-diseased and one non-infected macaque. The miRNA was enriched using a commercially available kit (Ambion) according to the supplier's instructions. Labeling of the microRNA was achieved by poly-uracil tailing and subsequent covalent linkage of a fluorescent dye (DY647, MoBiTec) according to a published protocol [14]. The labeled RNA was probed on microarray slides (Codelink, Amersham) spotted with 352 unique antisense miRNA oligonucleotides covering a wide range of currently known miRNA sequences from rats, mice, and humans (probeset 1564V1, Ambion). Microarrays were processed according to published protocols [15]. The fluorescence data were generated according to the MIAME guidelines and deposited in the Gene Expression Omnibus (GEO) database at  (accession number GSE12651).

To date, no miRNA-expression profile of brain from cynomolgus macaques has been published. Therefore, we first examined the basal miRNA expression and compared it to that of human brain. Correlation of the endogenous macaque miRNA expression pattern with published human brain miRNA profiles [16-18] revealed that the microarray setup was suitable for miRNA profiling in macaque brain as a model for human miRNA expression (additional file 1). We therefore assumed that BSE infection of cynomolgus macaques represents an appropriate model to predict miRNA regulation in acquired human CJD.

Thus, we performed a miRNA microarray analysis of brain samples derived from a BSE-infected and a non-infected macaque. Alterations in miRNA expression levels were only examined *post mortem*. Earlier stages could not be assessed due to the very limited number of experimental animals. Analysis was performed in a two-color dye swap design with six technical replicates. We identified 21 out of 275 human miRNAs that were up- or downregulated more than twofold. Differential expression was detected for both, high and low abundant miRNAs (Table 1). We excluded those miRNAs from further analysis showing divergent endogenous expression levels as compared to human brain, e.g. hsa-miR-103 and -107 (additional file 2) and the abundantly expressed members of the let-7 family.

**Table 1:** Differentially expressed miRNAs in the brain of BSE-infected macaques and their relative abundance in the *basis pontis *region of macaque brain

**Regulated miRNAs identified by microarray**	**relative abundance in macaque brain** [% of highest expressed miRNA]	**regulation factor**	
		
	**microarray**	**stem-loop qRT-PCR**	**Poly(A)-tailed qRT-PCR**
hsa-miR-26a	83,4	3.0 **	1.0
hsa-miR-30a-5p	1,2		
hsa-miR-30d	10,6		
hsa-miR-103	27,1		
hsa-miR-106b	9,0		
hsa-miR-107	41,3		
hsa-miR-124a	58,4	2.2	
hsa-miR-125a	23,6		
hsa-miR-128a	22,8		
hsa-miR-132	11,3		
hsa-miR-143	8,2	0.5	
hsa-miR-145	13,9	0.6	
hsa-miR-181a	26,3		
hsa-miR-191	13,9		
hsa-miR-195	4,9		
hsa-miR-219	1,3		
hsa-miR-320	6,9		
hsa-miR-342-3p	8,0	2.7 ***	1.13 ***
hsa-miR-361	8,7		
hsa-miR-490	3,6		
hsa-miR-494	2,1	3.7 ***	8.1 ***

** student t-test with Welch's correction; p < 0.01,

*** student t-test with Welch's correction; p < 0.001

**Figure 1:**
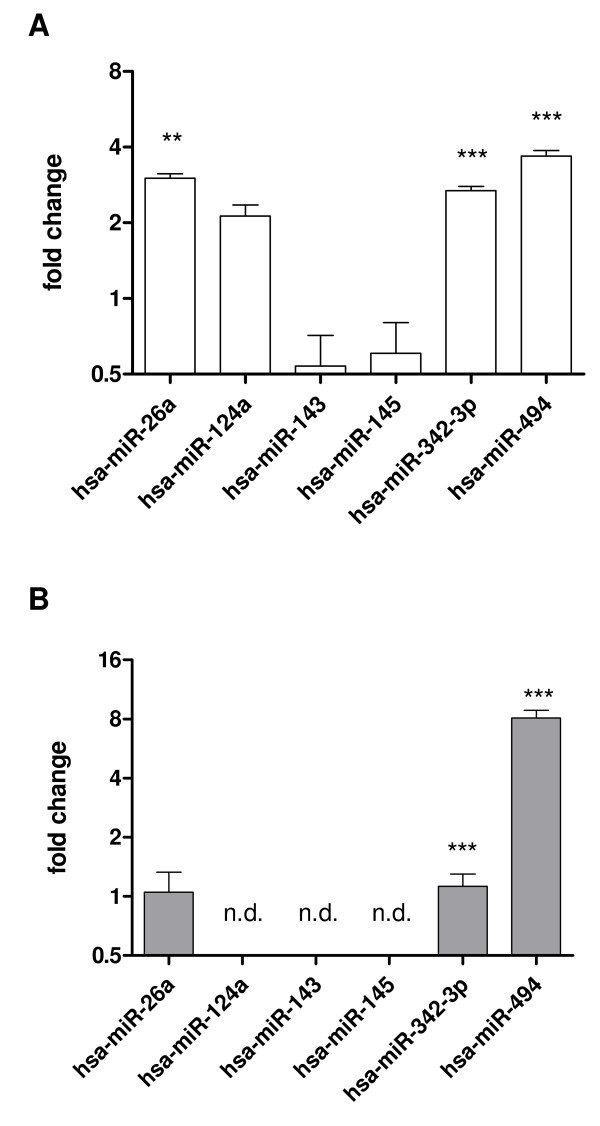
**Deregulated miRNAs in the brainof BSE-infected macaques identified by qRT-PCR analysis**. The *basis pontis *region of six BSE-infected and five non-infected macaques were dissected, miRNAs were isolated and 5 ng each analyzed for differential miRNA expression. **A**. MiRNA regulation according to stem-loop qRT-PCR (Applied Biosystems) and **B**. using poly(A)-tailed qRT-PCR. Analyses were performed twice in duplicate for each animal. Differential expression was analyzed by the ΔΔCT method [27] using the small nucleolar RNA RNU66 as a housekeeping RNA and the non-infected animals as calibrators. Statistical analysis was performed using unpaired t-test with Welch's correction (** p < 0.01, *** p < 0.001). n.d., not done. Shown are the mean regulation factors ± SD of all infected animals.

To validate our initial analysis we examined the regulation pattern in detail using a highly sensitive and quantitative approach. The relative expression of selected miRNA candidates hsa-miR-26a, hsa-miR-124a, hsa-miR-143, hsa-miR-145, hsa-miR-342-3p, and hsa-miR-494 were validated by quantitative reverse transcription PCR (qRT-PCR) analysis using a higher number of animals. MiRNA derived from brain samples of six BSE-infected cynomolgus macaques was compared to five age- and sex-matched controls. Five nanograms of the miRNA-enriched fraction derived from each of the eleven animals were used for stem-loop qRT-PCR according to the supplier's instructions (TaqMan MicroRNA Assay, Applied Biosystems) in a 7500 Real Time PCR System (Applied Biosystems). Individual samples were tested for the expression of the miRNA candidates in relation to the ubiquitously expressed small nucleolar RNA RNU66 that served as a housekeeping gene. Using this technique we could confirm the upregulation of hsa-miR-26a, -342-3p and -494 in the brain of six BSE-infected macaques compared to five non-infected controls (Figure 1A). The individual control animals showed comparable expression levels for each miRNA (additional file 3).

Since the mature forms of hsa-miR-342-3p and miR-494 reside on the 3'-arm of the pre-miRNA the stem-loop qRT-PCR cannot distinguish between DICER processed or unprocessed forms. In addition, the use of independent cDNA syntheses for each miRNA is likely to cause sample variation. Thus, we decided to use a second qRT-PCR-based assay (poly(A)-tailed qRT-PCR). The relative expression of microRNAs from each individual macaque was determined by poly(A)-tailing, cDNA-synthesis and subsequent qRT-PCR according to published procedures [19] with minor modifications. In brief, 100 ng of enriched miRNA fraction was polyadenylated according to the supplier's protocol (A-Plus™ Poly(A) Polymerase Tailing Kit, Epicentre) and reverse transcribed using a polyT-primer coupled to a unique sequence tag at its 5'-end. Quantitative reverse transcription PCR was performed with miRNA-specific forward primers for hsa-miR-26a, hsa-miR-342-3p, hsa-miR-494 or the small nucleolar RNA RNU66, respectively, and a universal reverse primer identical to the cDNA sequence tag (Figure 1B). Significant upregulation of hsa-miR-26a in BSE-infected macaques could not be confirmed using this independent method. However, our analysis revealed that both miRNAs, hsa-miR-342-3p and hsa-miR-494, were significantly upregulated in BSE-infected macaques (Table 1). Therefore, we could show that two miRNAs were upregulated in a non-human primate model for human prion disease.

Since miRNAs regulate their respective target genes via binding to specific seed regions in the 3'-UTR we examined the set of bioinformatically predicted mRNA-targets using a public prediction program (TargetScan, [20]). Beside others, putative target genes involved in neurodegenerative diseases were found for both, hsa-miR-342-3p and hsa-miR-494. Those genes are involved in protein aggregation disorders, such as tauopathies, Chorea Huntington, and spinocerebellar ataxia (additional file 4). We also perceived that hsa-miR-494 is deregulated in different types of cancer [21-23], and upregulated in a rat model for type 2 diabetes [24].

**Figure 2:**
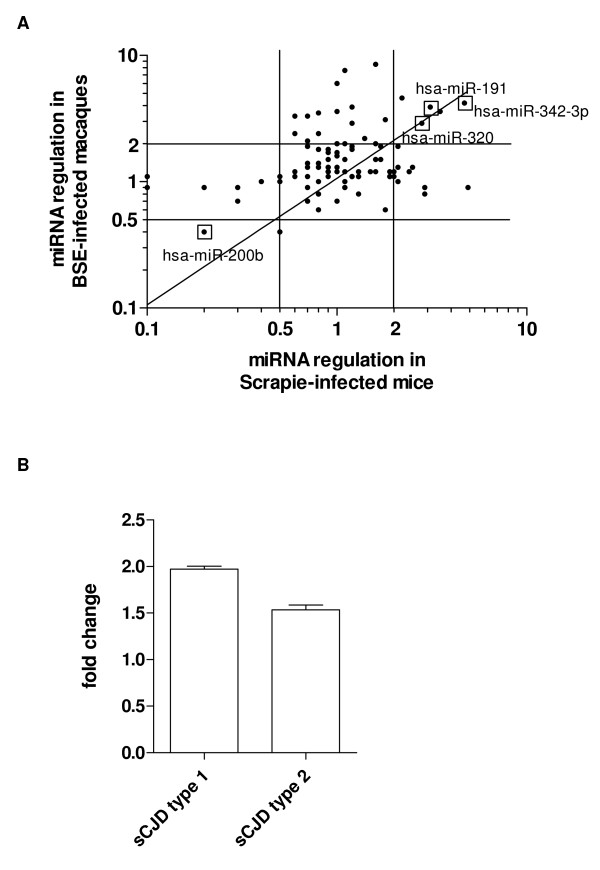
**Regulation of miRNA hsa-miR-342-3p in different prion disorders**. **A**. Regulation of miRNAs upon BSE infection in cynomolgus macaques compared to the published miRNA profile of Scrapie infection in mice. 500 ng miRNA enriched fraction from a BSE-infected and a non-infected rhesus macaque were fluorescently labeled and applied to miRNA microarray (Probeset 1564V1, Ambion). Regulation of each miRNA was calculated from the relative fluorescence values of the infected versus the non-infected animal. Acquired miRNA regulation profile was compared to a published profile derived from Scrapie-infected mice [11]. Correlation of both regulation profiles was statistically significant (Spearman correlation, p < 0.05). Relevant miRNAs regulated above twofold in both arrays are marked by an open square. **B**. Regulation of miR-342-3p in samples derived from brains of patients with type 1 sCJD, type 2 sCJD and a healthy control, respectively. 5 ng miRNA were applied to qRT-PCR specific for hsa-miR-342-3p (stem-loop qRT-PCR, Applied Biosystems). Relative miRNA expression was analyzed by ΔΔC_T _method [27] using the small nucleolar RNA RNU48 as a housekeeping RNA and the healthy human control as a calibrator. Statistical significance was determined by student-t-test (non-parametric with Welch's correction, *** p < 0.001). The mean regulation factor ± SD of duplicates from two independent experiments are shown.

Most intriguingly, Saba and colleagues examined the miRNA profile of Scrapie-infected mice [11]. In order to determine coincidences in the miRNA regulation patterns of both, Scrapie infection in mice and BSE infection in non-human primates, we correlated the miRNA expression patterns derived by miRNA-microarray. Even though several miRNAs were regulated in only one of both infection models the miRNA expression profiles showed a statistical significant correlation (Spearman, p < 0.05; Figure 2A). Among others, such as hsa-miR-191, hsa-miR-200b, hsa-miR-320 and several members of let-7 family we found that hsa-miR-342-3p was regulated at a late stage of disease in both, Scrapie-infected mice and BSE-infected macaques. It should be noted that only a very small region of the brain, i.e. *basis pontis*, was used for our analysis in non-human primates as compared to whole brain of Scrapie-infected mice. This may be reflected by the detected differences in regulation patterns. However, the comparable regulation of hsa-miR-342-3p may apply to prion disease in general. We concluded that upregulation of this particular miRNA may be a general marker candidate for late-stage prion disease or neurodegeneration. In this case hsa-miR-342-3p would also be upregulated in human prion disease. To strengthen our hypothesis we assessed the regulation of miR-342-3p in brains of sporadic Creutzfeldt-Jakob disease (sCJD) patients. In contrast to the experimentally induced prion disease in macaques, human sCJD occurs idiopathically. Depending on the age of onset, disease course, plaque formation, affected brain regions and biochemical properties of the prion protein aggregates human sCJD has been classified into two different types [25]. The miR-342-3p expression in the brains of a sCJD type 1 and a sCJD type 2 patient compared to a non-infected individual was analysed by qRT-PCR. MiRNA was enriched as described for simian brains and applied to stem-loop qRT-PCR for hsa-miR-342-3p. Data were normalized against the ubiquitously expressed small nucleolar RNA RNU48. Our pilot study revealed that hsa-miR-342-3p was upregulated approximately 2-fold in sCJD type 1 and 1.5-fold in sCJD type 2, respectively (Figure 2B) at a comparable level as in BSE-infected macaques.

Although regulation of hsa-miR-342-3p was not observed in miRNA studies using brain samples from Alzheimer's [26], or Huntington's disease [9] patients, we cannot rule out that this miRNA is not exclusively upregulated in prion-induced disorders. Thus the suitability of hsa-miR-342-3p as a novel biomarker for TSEs in animals and humans has to be further investigated. Future studies also have to reveal whether hsa-miR-494 is also upregulated in other prion disorders.

The presented conclusions rely on a considerable, but low number of experimental animals. In addition, we only assessed the expression of hsa-miR-342-3p in two sCJD patients which was compared to one healthy control. However, given that hsa-miR-342-3p was found in two experimental animal models and in human Creutzfeldt-Jakob disease, we assume that this miRNA may play a general role in the regulation of multiple target genes in late-stage prion disease. Analysis of the respective target genes in the central nervous system might be used as a tool to gain new insights in the molecular mechanism of neuronal decay.

## Abbreviations

BSE: bovine spongiform encephalopathy; cDNA: complementary DNA; miRNA: microRNA; PrP: prion protein; PrP^c^: cellular prion protein; PrP^Sc^: Scrapie associated prion protein; qRT-PCR: quantitative reverse transcription polymerase chain reaction; RISC: RNA induced silencing complex; sCJD: sporadic Creutzfeldt-Jakob disease; TSE: transmissible spongiform encephalopathy

## Conflicts of interests

The authors declare that they have no competing interests.

## Authors' contributions

This project was supported by the EU grant QLK1-CT-2002-01096 (to G.H.). All experiments were conceived and designed by J.M. and D.M.; R.H. and L.O. designed and evaluated microarrays. W.S. performed diagnosis of BSE infection in macaques. J.M. conceived and performed qRT-PCR and microarray. D.M. and J.M. performed target analysis. The manuscript was written by D.M., G.H., and J.M. with comments from all authors.

## Supplementary Material

Additional file 1 **Profile of miRNA expression in cynomolgus macaque brain**. Comparison of the miRNA expression profiles in the brain of cynomolgus macaques and published expression patterns of human brain. Click here for file

Additional file 2 **Relative abundance of miRNAs in human and macaque brain**. Comparison of the relative abundance of miRNAs that are differentially expressed in macaque brain upon BSE-infection to the abundance in human brain. Analysis was accomplished using published miRNA-expression profiles from human brain derived by microarray, qRT-PCR, and a cloning strategy, respectively. Click here for file

Additional file 3 **C_T_-values from qRT-PCR analysis of miRNA regulation upon BSE-infection**. C_T_-values derived from 4 independent qRT-PCR experiments comparing the expression of miRNAs hsa-miR-26a, hsa-miR-124a, hsa-miR-143, hsa-miR-145, hsa-miR-342-3p, and hsa-miR-494 in BSE-infected vs. non-infected cynomolgus macaques. Click here for file

Additional file 4 **Analysis of predicted targets for hsa-miR-342-3p and hsa-miR-494**. Analysis of the target predictions for hsa-miR-342-3p and hsa-miR-494 as revealed by the public target prediction program TargetScan (release 5.1, April 2009) for involvement in neurodegeneration and neurodegenerative disorders. Click here for file
