# Supplementary material for: Upregulation of miRNA hsa-miR-342-3p in experimental and idiopathic prion disease
Source: Mol Neurodegener. 2009 Aug 27;4:36. doi: 10.1186/1750-1326-4-36 (PMC2743691; doi:10.1186/1750-1326-4-36)
Supplement: Additional file 1 — Profile of miRNA expression in cynomolgus macaque brain. Comparison of the miRNA expression profiles in the brain of cynomolgus macaques and published expression patterns of human brain. [file 1750-1326-4-36-S1.pdf]

Additional file 1: Profile of miRNA expression in cynomolgus macaque brain

We analyzed the basal expression pattern of miRNAs in the brain *Macaca fascicularis* using miRNA microarrays and compared it to published miRNA profiles of human brain [1-3]. Shock-frozen brain tissue slides from a cynomolgus macaque were used for macro-dissection of distinct brain regions. The miRNA was enriched using a commercially available kit (mirVANA, Ambion) according to the supplier's instructions. Labelling of the microRNA fraction was achieved by poly-uracil tailing and subsequent covalent linkage of a fluorescent dye (DY647, MoBiTec, Goettingen, Germany) according to a published protocol [4]. The labelled RNA was probed on microarray slides (Amersham Codelink) that were spotted with 352 unique antisense miRNA oligos derived from rat, mouse and humans (Ambion probeset 1564V1) according to published procedures [5]. Expression data were acquired as relative fluorescence units.

The microarray based monkey brain signature was compared to the known miRNA expression patterns in humans analysed by miRNA microarray [1], qRT-PCR [2], or a cloning approach [3]. Our analysis revealed a highly significant correlation for all assessed data (Spearman, $p<0.0001$), independent of the methods by which the expression data had been acquired (Figure S1). These correlations give strong evidence that the overall miRNA expression pattern in the brain of cynomolgus macaques is comparable to the human miRNA signature. The miRNA microarray should therefore be suitable to identify regulated miRNAs in brains of BSE-infected macaques that should be predictive for human CJD.

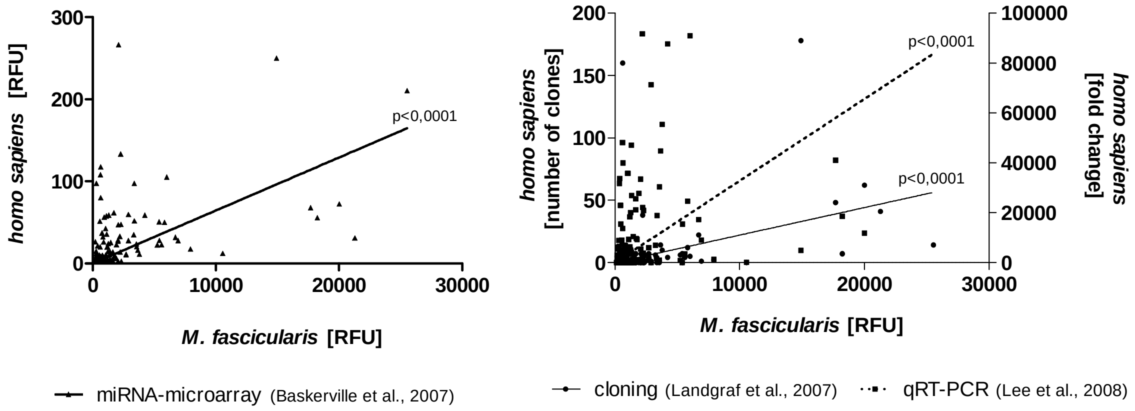

Figure S1: Correlation of miRNA expression data derived from human and simian brain

The miRNA fraction was isolated form shock frozen brain of cynomolgus macaques, 500 ng were fluorescently labelled and applied to a miRNA-microarray (Ambion V1.2). The mean of fluorescence for each miRNA derived from three independent arrays was compared to published miRNA expression data for human brain **A.** acquired by miRNA microarray [1] or **B.** by cloning [3] and qRT-PCR [2]. Comparison of the expression data from macaque and human brain revealed a significant correlation (Spearman, $p < 0.0001$).

References

1. Baskerville S, Bartel DP: **Microarray profiling of microRNAs reveals frequent coexpression with neighboring miRNAs and host genes.** *Rna* 2005, **11:**241-247.
2. Lee EJ, Baek M, Gusev Y, Brackett DJ, Nuovo GJ, Schmittgen TD: **Systematic evaluation of microRNA processing patterns in tissues, cell lines, and tumors.** *Rna* 2008, **14:**35-42.
3. Landgraf P, Rusu M, Sheridan R, Sewer A, Iovino N, Aravin A, Pfeffer S, Rice A, Kamphorst AO, Landthaler M, et al: **A mammalian microRNA expression atlas based on small RNA library sequencing.** *Cell* 2007, **129:**1401-1414.
4. Shingara J, Keiger K, Shelton J, Laosinchai-Wolf W, Powers P, Conrad R, Brown D, Labourier E: **An optimized isolation and labeling platform for accurate microRNA expression profiling.** *Rna* 2005, **11:**1461-1470.
5. Landgrebe J, Welzl G, Metz T, van Gaalen MM, Ropers H, Wurst W, Holsboer F: **Molecular characterisation of antidepressant effects in the mouse brain using gene expression profiling.** *J Psychiatr Res* 2002, **36:**119-129.
