# Supplementary material for: Upregulation of miRNA hsa-miR-342-3p in experimental and idiopathic prion disease
Source: Mol Neurodegener. 2009 Aug 27;4:36. doi: 10.1186/1750-1326-4-36 (PMC2743691; doi:10.1186/1750-1326-4-36)
Supplement: Additional file 2 — Relative abundance of miRNAs in human and macaque brain. Comparison of the relative abundance of miRNAs that are differentially expressed in macaque brain upon BSE-infection to the abundance in human brain. Analysis was accomplished using published miRNA-expression profiles from human brain derived by microarray, qRT-PCR, and a cloning strategy, respectively. [file 1750-1326-4-36-S2.pdf]

Additional file 2: Relative abundance of miRNAs in human and macaque brain

We found 21 miRNAs that were regulated in the brains of BSE-infected cynomolgus macaques. MiRNAs showing fluorescence values above the mean of all fluorescence values adding twice the standard deviation were defined to be robustly expressed. Only those miRNAs were included in the analysis showing robust expression in at least the infected or the non-infected state, respectively. Differential expression of individual miRNAs was assessed by the ratio of fluorescence signals of the BSE-infected animal versus the non-infected control. For further analysis a stringent threshold of an at least two-fold differential expression level was applied.

The endogenous expression of the regulated miRNAs were compared to the published relative expression profiles in brains of healthy humans acquired by miRNA microarray [1], cloning strategies [2], and qRT-PCR [3]. The relative abundance was normalized against the highest expression value detected for each assay. For our miRNA microarray the threshold for expression corresponds to a fluorescence of 7.5% of the highest expressed miRNA. The miRNAs were divided into high abundant ones that showed a minimum of three fold higher fluorescence compared to the threshold (> 22.5%) and low abundant miRNAs (< 22.5%). These base lines were also applied to the relative expression data of the assays used for comparison. Abundances were ranged accordingly and are displayed in table S1. The abundance was defined to be comparable if at least half of the assays showed the same abundance according to the base lines of expression in the brain of humans and macaques. We found accordant expression in all but three miRNAs (hsa-miR-103, hsa-miR-107, and hsa-miR-181a).

Table S1: Relative abundance of miRNAs in macaque and human brain

	relative abundance of miRNA in brain (percent of highest expressed miRNA)			
	m. fascicularis	*h. sapiens*		
identified by / miRNA	microarray	cloning [2]	qRT-PCR [3]	microarray [1]
hsa-miR-26a	83,4 %	23,0 %	n.d.	6,2 %
hsa-miR-30a-5p	1,2 %	1,1 %	7,1 %	0,8 %
hsa-miR-30d	10,6 %	3,9 %	4,8 %	2,2 %
hsa-miR-103	27,1 %	0,6 %	7,3 %	5,6 %
hsa-miR-106b	9,0 %	0,0 %	2,2 %	0,6 %
hsa-miR-107	41,3 %	0,0 %	0,1 %	2,4 %
hsa-miR-124a	58,4 %	100,0 %	3,9 %	50,0 %
hsa-miR-125a	23,6 %	2,8 %	72,9 %	21,0 %
hsa-miR-128a	22,8 %	6,7 %	19,7 %	10,0 %
hsa-miR-132	11,3 %	0,0 %	0,1 %	5,6 %
hsa-miR-143	8,2 %	3,9 %	0,4 %	53,4 %
hsa-miR-145	13,9 %	0,0 %	0,8 %	4,8 %
hsa-miR-181a	26,3 %	12,4 %	13,8 %	6,4 %
hsa-miR-191	13,9 %	0,6 %	24,4 %	4,2 %
hsa-miR-195	4,9 %	6,2 %	16,1 %	4,8 %
hsa-miR-219	1,3 %	0,0 %	0,2 %	2,2 %
hsa-miR-320	6,9 %	0,6 %	7,4 %	1,8 %
hsa-miR-342-3p	8,0 %	0,6 %	26,8 %	5,6 %
hsa-miR-361	8,7 %	0,0 %	2,0 %	n.d.
hsa-miR-490	3,6 %	0,0 %	n.d.	n.d.
hsa-miR-494	2,1 %	0,0 %	n.d.	n.d.

References

1. Baskerville S, Bartel DP: **Microarray profiling of microRNAs reveals frequent coexpression with neighboring miRNAs and host genes.** *Rna* 2005, **11:**241-247.
2. Landgraf P, Rusu M, Sheridan R, Sewer A, Iovino N, Aravin A, Pfeffer S, Rice A, Kamphorst AO, Landthaler M, et al: **A mammalian microRNA expression atlas based on small RNA library sequencing.** *Cell* 2007, **129:**1401-1414.
3. Lee EJ, Baek M, Gusev Y, Brackett DJ, Nuovo GJ, Schmittgen TD: **Systematic evaluation of microRNA processing patterns in tissues, cell lines, and tumors.** *Rna* 2008, **14:**35-42.
