# Supplementary material for: Upregulation of miRNA hsa-miR-342-3p in experimental and idiopathic prion disease
Source: Mol Neurodegener. 2009 Aug 27;4:36. doi: 10.1186/1750-1326-4-36 (PMC2743691; doi:10.1186/1750-1326-4-36)
Supplement: Additional file 3 — CT-values from qRT-PCR analysis of miRNA regulation upon BSE-infection. CT-values derived from 4 independent qRT-PCR experiments comparing the expression of miRNAs hsa-miR-26a, hsa-miR-124a, hsa-miR-143, hsa-miR-145, hsa-miR-342-3p, and hsa-miR-494 in BSE-infected vs. non-infected cynomolgus macaques. [file 1750-1326-4-36-S3.pdf]

Additional file 3: Analysis of predicted targets for hsa-miR-342-3p and hsa-miR-494

The public target prediction program TargetScan (release 5.1, April 2009) [1, 2] was used for analysis of potential targets of hsa-miR-342-3p and hsa-miR-494. Even though target prediction programs have been proven to miss several targets the TargetScan algorithm was shown to provide the highest accuracy [3]. The targets are listed with their gene name according to the TargetScan program. Genes were examined systematically for the function and linkage to prion diseases, neuronal metabolism, neurodegeneration, and other neuronal disorders by pubmed database search using the keywords “prion, neuron, neuronal, neurodegeneration, Alzheimer*, Huntington*” in conjunction with the official gene name and their aliases, respectively.

1) refers to the gene name used in the relevant citation. 2) Online Mendelian Inheritance in Man (OMIM) entry. Johns Hopkins University, Baltimore, MD. http://www.ncbi.nlm.gov/omim
AD: Alzheimer’s disease, HD: Huntington’s disease, PD: Parkinson’s disease, PrP: Prion protein, SCA7: Spinocerebellar ataxia 7

Supplemental table 2: Predicted target genes for BSE-upregulated hsa-miRNAs -342 and -494 linked to neurodegenerative disorders

**Official symbol**	**Relevant alias [1)]**	**Official name**	**Targeted by hsa-miR-**	**Relevant citation**	**Neurodegenera-tive disorder**	**OMIM [2)]**
ATRX		alpha thalassemia/mental retardation syndrome X-linked	494	[4]	X-linked mental retardation	*300032, #309580
ATXN7		ataxin 7	342	[5]	SCA7	
CGGBP1		CGG triplet repeat binding protein 1	494	[6]	fragile X-syndrome	
CUGBP2	ETR-3	CUG triplet repeat, RNA binding protein 2	494	[7]	taupathies	
CYFIP2		cytoplasmic FMR1 interacting protein 2	494	[8]	fragile X-syndrome	
CAST		Calpastatin	494	[9]	AD	
DNAJA2	RDJ2	DnaJ (Hsp40) homolog, subfamily A, member 2	494	[10]	PrP metabolism	
DPYSL2	CRMP2	dihydropyrimidinase-like 2	494	[11]	AD	
GRIK2	GluR6	glutamate receptor	494	[12]	HD	
HIP1		Huntingtin interacting protein 1	342	[13]	HD	*061767
JARID1C		jumonji, AT rich interactive domain 1C	494	[14]	X-linked mental retardation	*314690, #300534
KIAA2022		RP11-130N24.1	342, 494	[15]	X-linked mental retardation	*300560, #300263
LMO4		LIM domain only 4	494	[16]	AD	
LRP1B		low density lipoprotein-related protein 1B	494	[17]	AD	
MSI1		musashi homolog 1	342	[18]	taupathies	
NRF1	NRF-1	nuclear respiratory factor 1	342	[19]	fragile X-syndrome	*309550
PHF8		PHD finger protein 8	494	[20]	X-linked mental retardation	#30263
PPARGC1A	PGC-1alpha	peroxisome proliferator-activated receptor gamma	494	[21]	HD	
ROCK1		Rho-associated, coiled-coil containing protein kinase 1	494	[22]	AD	*104760
SFRS7	9G8	splicing factor, arginine/serine-rich 7, 35kDa	494	[23]	taupathies	
SFRS8	SWAP	splicing factor, arginine/serine-rich 8	494	[24]	taupathies	
SIRT1		sirtuin	494	[25] [26]	AD, fragile X-syndrome	
TTBK2		tau tubulin kinase 2	342	[27]	taupathies	
UBE2K		ubiquitin-conjugating enzyme E2K	494	[28]	HD	
XIAP		X-linked inhibitor of apoptosis	494	[29]	PD	

References:

1. Grimson A, Farh KK, Johnston WK, Garrett-Engele P, Lim LP, Bartel DP: **MicroRNA targeting specificity in mammals: determinants beyond seed pairing.** *Mol Cell* 2007, **27:**91-105.
2. Lewis BP, Shih IH, Jones-Rhoades MW, Bartel DP, Burge CB: **Prediction of mammalian microRNA targets.** *Cell* 2003, **115:**787-798.
3. Hendrickson DG, Hogan DJ, Herschlag D, Ferrell JE, Brown PO: **Systematic identification of mRNAs recruited to argonaute 2 by specific microRNAs and corresponding changes in transcript abundance.** *PLoS ONE* 2008, **3:**e2126.
4. Wada T, Fukushima Y, Saitoh S: **A new detection method for ATRX gene mutations using a mismatch-specific endonuclease.** *Am J Med Genet A* 2006, **140:**1519-1523.
5. Hara K, Momose Y, Tokiguchi S, Shimohata M, Terajima K, Onodera O, Kakita A, Yamada M, Takahashi H, Hirasawa M, et al: **Multiplex families with multiple system atrophy.** *Arch Neurol* 2007, **64:**545-551.
6. Muller-Hartmann H, Deissler H, Naumann F, Schmitz B, Schroer J, Doerfler W: **The human 20-kDa 5'-(CGG)(n)-3'-binding protein is targeted to the nucleus and affects the activity of the FMR1 promoter.** *J Biol Chem* 2000, **275:**6447-6452.
7. Leroy O, Dhaenens CM, Schraen-Maschke S, Belarbi K, Delacourte A, Andreadis A, Sablonniere B, Buee L, Sergeant N, Caillet-Boudin ML: **ETR-3 represses Tau exons 2/3 inclusion, a splicing event abnormally enhanced in myotonic dystrophy type I.** *J Neurosci Res* 2006, **84:**852-859.
8. Schenck A, Bardoni B, Moro A, Bagni C, Mandel JL: **A highly conserved protein family interacting with the fragile X mental retardation protein (FMRP) and displaying selective interactions with FMRP-related proteins FXR1P and FXR2P.** *Proc Natl Acad Sci U S A* 2001, **98:**8844-8849.
9. Rao MV, Mohan PS, Peterhoff CM, Yang DS, Schmidt SD, Stavrides PH, Campbell J, Chen Y, Jiang Y, Paskevich PA, et al: **Marked calpastatin (CAST) depletion in Alzheimer's disease accelerates cytoskeleton disruption and neurodegeneration: neuroprotection by CAST overexpression.** *J Neurosci* 2008, **28:**12241-12254.
10. Beck KE, Kay JG, Braun JE: **Rdj2, a J protein family member, interacts with cellular prion PrP(C).** *Biochem Biophys Res Commun* 2006, **346:**866-871.
11. Cole AR, Noble W, van Aalten L, Plattner F, Meimaridou R, Hogan D, Taylor M, LaFrancois J, Gunn-Moore F, Verkhratsky A, et al: **Collapsin response mediator protein-2 hyperphosphorylation is an early event in Alzheimer's disease progression.** *J Neurochem* 2007, **103:**1132-1144.
12. Zeng W, Gillis T, Hakky M, Djousse L, Myers RH, MacDonald ME, Gusella JF: **Genetic analysis of the GRIK2 modifier effect in Huntington's disease.** *BMC Neurosci* 2006, **7:**62.
13. Bhattacharyya NP, Banerjee M, Majumder P: **Huntington's disease: roles of huntingtin-interacting protein 1 (HIP-1) and its molecular partner HIPPI in the regulation of apoptosis and transcription.** *Febs J* 2008, **275:**4271-4279.
14. Jensen LR, Amende M, Gurok U, Moser B, Gimmel V, Tzschach A, Janecke AR, Tariverdian G, Chelly J, Fryns JP, et al: **Mutations in the JARID1C gene, which is involved in transcriptional regulation and chromatin remodeling, cause X-linked mental retardation.** *Am J Hum Genet* 2005, **76:**227-236.
15. Cantagrel V, Lossi AM, Boulanger S, Depetris D, Mattei MG, Gecz J, Schwartz CE, Van Maldergem L, Villard L: **Disruption of a new X linked gene highly expressed in brain in a family with two mentally retarded males.** *J Med Genet* 2004, **41:**736-742.

16. Leuba G, Vernay A, Vu D, Walzer C, Belloir B, Kraftsik R, Bouras C, Savioz A: **Differential expression of LMO4 protein in Alzheimer's disease.** *Neuropathol Appl Neurobiol* 2004, **30:**57-69.
17. Jaeger S, Pietrzik CU: **Functional role of lipoprotein receptors in Alzheimer's disease.** *Curr Alzheimer Res* 2008, **5:**15-25.
18. Cuadrado A, Garcia-Fernandez LF, Imai T, Okano H, Munoz A: **Regulation of tau RNA maturation by thyroid hormone is mediated by the neural RNA-binding protein musashi-1.** *Mol Cell Neurosci* 2002, **20:**198-210.
19. Kumari D, Gabrielian A, Wheeler D, Usdin K: **The roles of Sp1, Sp3, USF1/USF2 and NRF-1 in the regulation and three-dimensional structure of the Fragile X mental retardation gene promoter.** *Biochem J* 2005, **386:**297-303.
20. Laumonnier F, Holbert S, Ronce N, Faravelli F, Lenzner S, Schwartz CE, Lespinasse J, Van Esch H, Lacombe D, Goizet C, et al: **Mutations in PHF8 are associated with X linked mental retardation and cleft lip/cleft palate.** *J Med Genet* 2005, **42:**780-786.
21. Weydt P, Pineda VV, Torrence AE, Libby RT, Satterfield TF, Lazarowski ER, Gilbert ML, Morton GJ, Bammler TK, Strand AD, et al: **Thermoregulatory and metabolic defects in Huntington's disease transgenic mice implicate PGC-1alpha in Huntington's disease neurodegeneration.** *Cell Metab* 2006, **4:**349-362.
22. Pedrini S, Carter TL, Prendergast G, Petanceska S, Ehrlich ME, Gandy S: **Modulation of statin-activated shedding of Alzheimer APP ectodomain by ROCK.** *PLoS Med* 2005, **2:**e18.
23. Gao L, Wang J, Wang Y, Andreadis A: **SR protein 9G8 modulates splicing of tau exon 10 via its proximal downstream intron, a clustering region for frontotemporal dementia mutations.** *Mol Cell Neurosci* 2007, **34:**48-58.
24. Wang J, Gao QS, Wang Y, Lafyatis R, Stamm S, Andreadis A: **Tau exon 10, whose missplicing causes frontotemporal dementia, is regulated by an intricate interplay of cis elements and trans factors.** *J Neurochem* 2004, **88:**1078-1090.
25. Kim D, Nguyen MD, Dobbin MM, Fischer A, Sananbenesi F, Rodgers JT, Delalle I, Baur JA, Sui G, Armour SM, et al: **SIRT1 deacetylase protects against neurodegeneration in models for Alzheimer's disease and amyotrophic lateral sclerosis.** *Embo J* 2007, **26:**3169-3179.
26. Biacsi R, Kumari D, Usdin K: **SIRT1 Inhibition Alleviates Gene Silencing in Fragile X Mental Retardation Syndrome.** *PLoS Genet* 2008, **4:**e1000017.
27. Houlden H, Johnson J, Gardner-Thorpe C, Lashley T, Hernandez D, Worth P, Singleton AB, Hilton DA, Holton J, Revesz T, et al: **Mutations in TTBK2, encoding a kinase implicated in tau phosphorylation, segregate with spinocerebellar ataxia type 11.** *Nat Genet* 2007, **39:**1434-1436.
28. de Pril R, Fischer DF, Roos RA, van Leeuwen FW: **Ubiquitin-conjugating enzyme E2-25K increases aggregate formation and cell death in polyglutamine diseases.** *Mol Cell Neurosci* 2007, **34:**10-19.
29. Tsang AH, Lee YI, Ko HS, Savitt JM, Pletnikova O, Troncoso JC, Dawson VL, Dawson TM, Chung KK: **S-nitrosylation of XIAP compromises neuronal survival in Parkinson's disease.** *Proc Natl Acad Sci U S A* 2009, **106:**4900-4905.
